# Validity of the Pneumonitor for Analysis of Short-Term Heart Rate Asymmetry Extended with Respiratory Data in Pediatric Cardiac Patients

**DOI:** 10.3390/jcm13164654

**Published:** 2024-08-08

**Authors:** Jakub S. Gąsior, Marcel Młyńczak, Maciej Rosoł, Piotr Wieniawski, Radosław Pietrzak, Bożena Werner

**Affiliations:** 1 Department of Pediatric Cardiology and General Pediatrics, Medical University of Warsaw, 02-091 Warsaw, Poland; 2 Institute of Metrology and Biomedical Engineering, Warsaw University of Technology, 02-525 Warsaw, Poland

**Keywords:** heart rate asymmetry, Pneumonitor, respiratory rate, pediatric cardiology

## Abstract

**Background**: Wearable technologies have been developed to measure physiological parameters conveniently. To consider the new measurement device valid, the crucial point is to assess its reliability with the gold standard. The study aimed to assess the validity of the Pneumonitor (PM, fs = 250 Hz) for acquisition of 5 min RR intervals (RRi) for analysis of heart rate asymmetry (HRA) in relation to the electrocardiography (ECG, fs = 1000 Hz) in a group of 19 pediatric cardiac patients. Association between HRA and respiratory rate (RespRate) was verified. **Methods**: The validation comprised Bland–Altman analysis, intraclass correlation coefficient, and Student’s *t*-test. **Results**: Sufficient agreement between 10 from 16 HRA parameters was observed. Different HRA parameters values calculated based on RRi from both devices were related to different results of correlation analysis between two parameters and RespRate. **Conclusions**: The PM might be considered valid for recording RRi, which are then processed to calculate selected HRA parameters in a group of pediatric cardiac patients in rest condition. However, RRi recorded using devices with fs < 250 Hz may be not adequate for reliable HRA analysis.

## 1. Introduction

Patients with congenital heart disease/defects (CHDs) present cardiac autonomic dysfunction [1]. Calculation of heart rate variability (HRV) parameters is a non-invasive approach to evaluate cardiac autonomic responsiveness [2]. Heart rate asymmetry (HRA), defined using different approaches using the Poincaré plot (PP), reflects imbalanced contribution of heart rate (HR) accelerations and decelerations to short-, long-term, and total HRV [3,4,5,6,7]. Authors of recent studies on asymmetric properties of HR microstructure indicate that the dependency of HRA on respiratory rate (RespRate) [8,9] and influence of sampling frequency (fs) of the recorded signal on HRA data [10] require more studies.

Over the last decade, new wearable digital health technologies have been developed to measure physiological parameters more readily [11]. The Pneumonitor is a compact, academically developed device which offers synchronized recording of RR intervals (RRi) via single-lead electrocardiography (ECG, fs = 250 Hz) and respiratory rate as well as tidal volume equivalent using the impedance pneumography (IP) technique utilizing the same set of electrodes [12]. The Pneumonitor is hence considered a wearable device allowing both respiratory and cardiac activity to be measured and cardiorespiratory coupling in various measurement conditions to be evaluated.

Pediatric cardiologists have rarely considered HRV analysis in the setting of pediatric patients with heart disease/defect [13]. The aim of this study was to assess the validity of the Pneumonitor for acquisition and analysis of short-term RRi in the context of HRA in comparison to the clinical reference ECG device (fs = 1000 Hz) in a group of pediatric cardiac patients. Furthermore, this study aimed also to analyze the association between HRA parameters and respiratory rate during stable conditions in this group.

## 2. Materials and Methods

### 2.1. Population

A total of 19 pediatric cardiac patients (of both sexes) participated in the study. The inclusion criteria were as follows: 7–18 years, absence of infection, and no change in medications in the last 3 months (in cases of constant pharmacological treatment). The study was approved by the University Bioethical Committee (KB/70/2021, 14 June 2021). All parents or legal guardians and patients 16 years old and older gave their informed written consent.

### 2.2. Procedures and Measurement Conditions

Patients and their parents/legal guardians were made aware conversationally of the study objectives, measurement protocol, potential risks involved, and its benefits. Recordings were performed between 8:30 am and 2:00 pm in a hospital room with stable, controlled temperature and humidity. Patients were advised to abstain from physical activity on the day before and the day of the study, avoid junk food, sugary drinks, and snacks, and to use the toilet (if necessary) before examinations. The examination was conducted at least one hour after breakfast [14].

### 2.3. RRi Data Acquisition Using an ECG and the Pneumonitor

For ECG recording, 10 electrodes were placed in standard positions. For the Pneumonitor (PM) measurement, 5 electrodes were positioned according to the scheme presented elsewhere [12]. RRi were recorded simultaneously using ECG (Custo cardio 100 12-channel PC ECG system; Custo med GmbH, Ottobrunn, Germany), and the PM, in the supine position for 5 min. The PM measured single-lead ECG signals along with IP with the same set of electrodes (standard Holter-type, disposable). For the PM, ECG signal pre-processing included the following: (i) baseline alignment; (ii) R peaks detection using Stationary Wavelet Transform [15]; (iii) manual correction of any inaccurately detected R peaks (if necessary, based on visual inspection); and (iv) estimation of RRi between successive R peaks. The IP signal was measured using the tetrapolar method with a specified electrode configuration [16].

### 2.4. Artifacts Identification and Correction

Registered ECGs were reviewed by a pediatric cardiologist to confirm sinus rhythm and identify any ectopic beats. The RRi were exported from the ECG software (Custo cardio 100 12-channel PC ECG system; Custo med GmbH, Ottobrunn, Germany), and analytical scripts were prepared for PM data to identify artifacts based on graphical presentation of raw RRi from both devices along with manual editing, according to recommendations [17]. Technical artifacts were identified as one of seven types of errors—correction was performed for T2-T5 and T6b artifacts. T1 and T6a artifacts were not corrected since it is not possible to identify both artifacts without simultaneous ECG recordings [17]. Physiological artifacts present in the ECG signal were replaced by interpolated RRi from adjacent RRi [17].

### 2.5. Heart Rate Asymmetry

To quantify HRA, Guzik and Piskorski analyses [3,4,5,6] and Porta’s index (PI) [7] were used (implemented in the HRAExplorer software, https://hraexplorer.com, accessed on 15 January 2024). Guzik and Piskorski proposed two areas of HRA analysis: (i) study of contributions (defined as the percentage of cumulative distance of the points) of accelerations (_a_) and decelerations (_d_) to short-term (SD1) and long-term (SD2) variability [3,4], and (ii) analysis of monotonic runs of accelerations (AR), decelerations (DR), and neutral (NR) [4]. For short-term variability, authors offered the following definitions: C1_a_ and C1_d_ (Guzik’s index) as relative contributions of accelerations (SD1_a_) and decelerations (SD1_d_), respectively, to short-term variance (SD1); and for long-term variability: C2_a_ and C2_d_ as relative contributions of accelerations (SD2_a_) and decelerations (SD2_d_), respectively, to long-term variance (SD2) [6]. HRA is considered to be present if contributions of HR decelerations to short-term variability are greater than those of accelerations (C1_d_ > C1_a_, i.e., C1_d_ > 0.50) and the contributions of accelerations to long-term variability are greater than those of decelerations (C2_a_ > C2_d_, i.e., C2_d_ < 0.50). The PI is based on the evaluation of the percentage of negative RRi (points below the line of identity in the PP) with respect to the number of overall points not on the line of identity. The PI < 50% means that decelerations in general are less numerous than accelerations [7]. Asymmetry in PP suggests that HR accelerations operate in a different manner than decelerations, possibly due to baroreflex responses [18]. Visual detection of narrowed and shortened parallels to the line of identity shape of the PP could indicate sympathetic predominance [19]. Recently, it has been suggested that Guzik’s index may assess vagal withdrawal rather than sympathetic activation during the tilt maneuver [20].

### 2.6. RespRate

RespRates were estimated as follows: (i) the raw IP signal was smoothed using a 1 s window to remove the cardiac component [21]; (ii) respiratory onsets were identified based on the differentiated, flow-related signal; (iii) RespRates were calculated between successive respiratory onsets. Impedance was not transformed into volume in liters, as it was assumed that impedance changes replicate the TV signal in terms of shape [22]. The volume of the first breath was assigned with a value of 1, and the volumes of all subsequent breaths were related to this initial value. Inspiratory and expiratory phases were detected from the differentiated signal, and inspiratory and expiratory TVs were estimated as the difference between the maximum after inspiration and the minimum before inspiration, and the maximum before expiration and the minimum after expiration, respectively.

### 2.7. Statistical Analysis

Agreement of parameters between the reference ECG and the Pneumonitor was assessed using a Bland–Altman plot with limits of agreement (LoA) [23] and the intraclass correlation coefficient (ICC) [24]. An agreement sufficient for the interchangeable use of the two methods is indicated when a lower bound of the 95% confidence interval (CI) exceeds 0.75 [25]. The smallest worthwhile change (SWC) was determined by multiplying the between-subject standard deviation of ECG signal values by 0.2, defining the maximum allowed difference between methods. The two methods are considered to be in agreement if the LoA do not exceed the SWC. Student’s *t*-test was employed to compare changes between parameters calculated based on RRi from ECG and PM. Pearson’s correlation coefficient was calculated to illustrate the association between HRA parameters and RespRate.

## 3. Results

### 3.1. Participants Characteristics

Results from three patients were excluded due to poor signal quality (n = 2) and unconfirmed diagnosis (n = 1). The analysis included results from 16 pediatric Polish Caucasian patients (6 girls), consisting of 5 with congenital heart disease, 4 with cardiac arrhythmia, and 7 with cardiomyopathy. The mean ± SD age, body mass, stature, body mass index, and RespRate were 13 ± 3 years, 58 ± 25 kg, 158 ± 18 cm, 22 ± 6 kg/m^2^, and 19 ± 5 breaths/min, respectively.

### 3.2. Agreement of HRA Parameters

**Figure 1:**
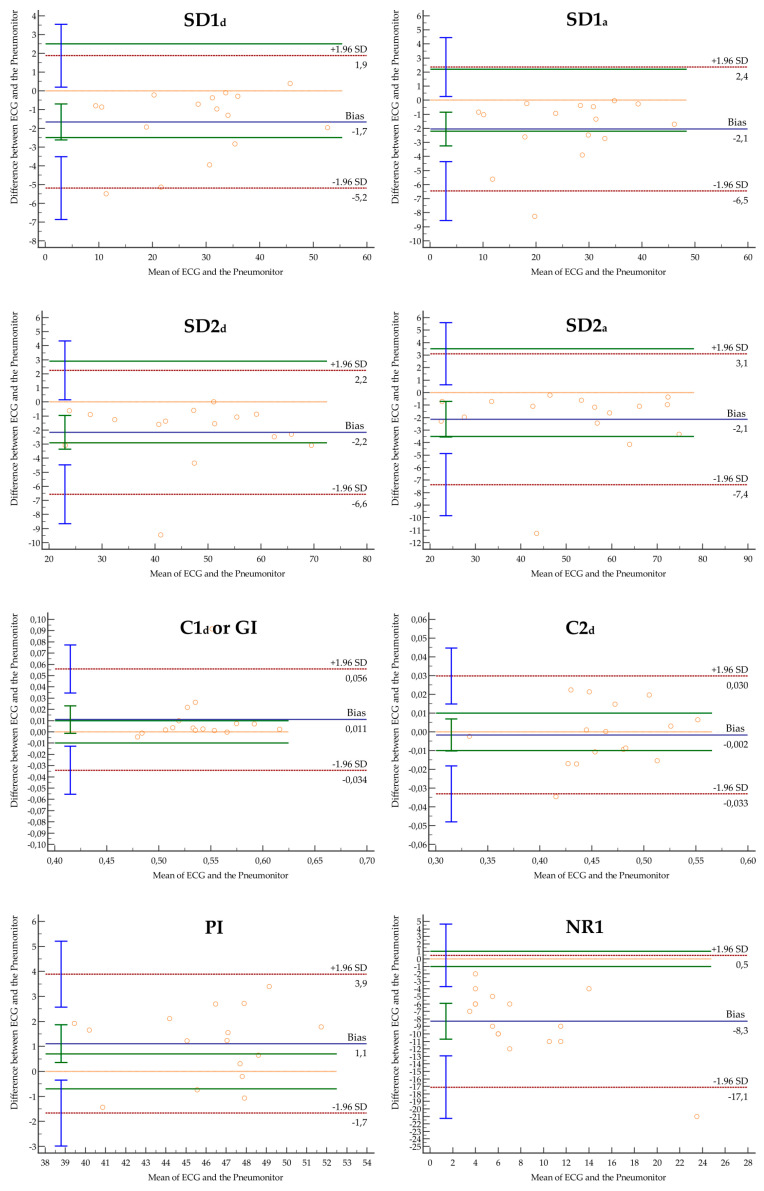
Bland–Altman plots for HRA parameters generated using RRi obtained from ECG and the Pneumonitor. The blue whiskers indicate the confidence intervals for the mean, while the green whiskers denote the LoA. The green lines represent the SWC.

Agreement statistics data for HRA parameters calculated based on RRi obtained using ECG and PM are shown in Table 1. A sufficient agreement was observed for SD1_d_, SD2_d_, SD2_a_, C2_d_, DR1, DR2, DR3, AR1, AR4, and AR5. The LoA exceeded the SWC between ECG and PM for all parameters. SWC and the number of patients for whom LoA exceeded the defined SWC (in brackets) were as follows: SD1_d_ = 2.5 (4), SD1_a_ = 2.2 (6), SD2_d_ = 2.9 (4), SD2_a_ = 3.5 (2), C1_d_, C2_d_ = 0.01 (3, 8), PI = 0.7 (12), DR1 = 3 (9), DR2 = 2 (7), DR3 = 1 (8), DR4 = 0.4 (7), DR5 = 0.2 (7), AR1 = 4 (8), AR2 = 3 (5), AR3 = 1 (6), AR4 = 1 (5), AR5 = 1 (2), NR1 = 1 (16), NR2 = 0.2 (4). There were significant differences between NR1 and NR2 calculated based on RRi obtained using different devices. The Bland–Altman plots are presented in Figure 1.

**Figure 2:**
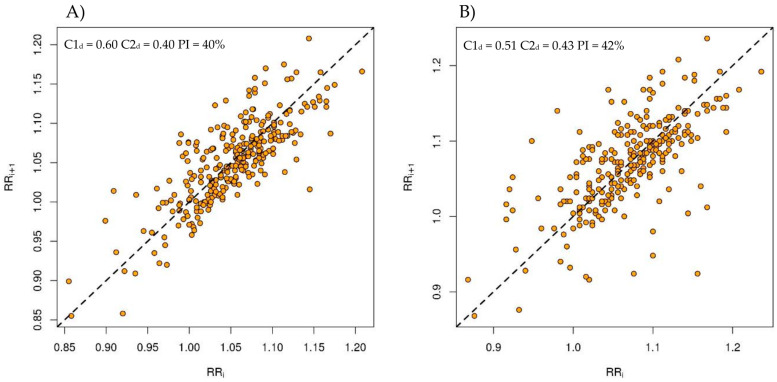
Poincaré plots with results of C1d, C2d, and PI from (**A**) ECG and (**B**) Pneumonitor of patient #6 (RespRate = 8 breaths/min).

Poincaré plots with results of C1_d_, C2_d_, and PI from ECG and PM for one of the patients (#6) with RespRate = 8 breaths/min are presented in Figure 2.

### 3.3. Correlation between HRA Parameters and RespRate

Correlation between HRA parameters calculated based on RRi from ECG and PM and RespRate are shown in Table 2. There was a significant correlation between RespRate and C1_d_, C2_d_, PI, DR1, AR1, AR2, AR4, and AR5, calculated based on RRi from ECG. There were differences in term of statistical significance of correlation analysis between RespRate and HRA parameters calculated based on RRi obtained using ECG and PM for C1_d_, C2_d_—there was a significant correlation between RespRate and those parameters calculated using ECG only.

## 4. Discussion

Sufficient agreement between following HRA parameters: SD1_d_, SD2_d_, SD2_a_, C2_d_, DR1, DR2, DR3, AR1, AR4, and AR5, calculated based on RRi acquired during rest, stable measurement conditions using the Pneumonitor and ECG was observed in pediatric cardiac patients. Importantly, insufficient agreement was observed for Guzik’s and Porta’s indexes, considered as the most popular HRA indexes. Different Guzik’s index values calculated based on RRi from the Pneumonitor and ECG were related to different results of correlation analysis between this parameter and respiratory rate. Devices with sampling frequency less than 250 Hz may be not adequate for reliable HRA analysis.

To consider the new measurement method/device/tool as interchangeable with another one (often gold standard method), the crucial point is to calculate the a priori acceptable LoA, to define the minimal agreement [23]. All analyzed parameters here showed a LoA that exceeded the defined a priori maximum acceptable difference (i.e., smallest worthwhile change—SWC). This is contradictory to the sufficiently high ICC values for selected HRA indices. Although the consideration of LoA < SWC represents an important criterion for agreement analysis, we believe it has a limitation. Since LoA is calculated from the standard deviation of differences between values obtained using different methods/devices, LoA will be low whenever the differences from all subjects tend to be homogeneous. In our opinion, more complete analysis would involve the comparison of LoA and SWC together with the one-sample *t*-test to check if the fixed bias is different from zero.

A series of consecutive RRi prolongations, shortenings, and with no changes in values represent HR deceleration runs (DR, e.g., a pair of decelerations—DR2), acceleration runs (AR, e.g., a run of three accelerations—AR3), and neutral runs (NR), respectively. However, it was underlined that NR are caused by a low sampling frequency [4,10] rather than the character of the sinus node activity as physiologically no consecutive two beats of the heart are the same [26]. Higher sampling frequency improves the precision of RRi measurement and consequently limits the number of neutral runs [10]. In our study, a significantly higher number of NR (consecutive RRi which have identical duration) were identified in RRi series from PM with fs = 250 Hz than from ECG with fs = 1000 Hz. In a study of the HR microstructure using 24 h ECGs sampled at a frequency of 200 Hz, the number of NR was up to 6–7%, whereas using the sampling frequency of 8000 Hz, the number of NR was less than 1% [4,27]. Information on sampling frequency but also on the number of NR should be reported in studies on the HRV.

The duration of cardiac cycles during a single breath varies, i.e., RRi are longer during expiration (grouped bradycardic runs) and shorter during inspiration (grouped tachycardic runs) [28,29]. Importantly, in healthy humans, the expiratory phase lasts longer during spontaneous breathing [4,30], and as the breathing rate increases, both the expiratory and inspiratory times are shortened [31]. The dependence of HRA on alterations in respiratory rate is under debate [8,9,31,32,33]. Guzik’s and Porta’s indexes significantly increase during a symmetrical breathing pattern (inspiration and expiration controlled in a 1:1 ratio) compared to a physiological pattern (1:2 ratio) in young healthy volunteers in the supine position at 0.22 Hz breathing [8]. This was confirmed for 0.25 Hz breathing in the sitting position [31]. A very recent study found that increased inspiratory duration and increased expiratory duration have a positive impact on the magnitudes of the HRA indexes in young healthy volunteers in the sitting position [32]. Precisely, the optimal combination for maximizing HRA indexes was found to be an inspiratory duration of 4 s and an expiratory duration of 6 s [32]. Although the differences between the HRA parameters calculated based on RRi series from PM and ECG were nominally small, it seems that they were related to different results of correlation analysis between some parameters and respiratory rate. Guzik’s index and relative contributions of decelerations to long-term variance calculated based on RRi from ECG were significantly correlated with respiratory rate, whereas those calculated based on RRi from PM were not. As is known, regularizing breathing at a well-tolerated rate might stimulate efferent asymmetric autonomic patterns directed to the heart and/or might induce asymmetric responses of reflex cardiac control circuits such as the baroreflex [9]. Reliable verification of changes in the cardiac autonomic nervous system modulation by the analysis of HR variability (HRV) during different breathing patterns is crucial in, e.g., HRV biofeedback applications [34].

HRV analysis requires RR series preprocessing [35]. Parameters from variance-based methods (e.g., time-domain, frequency-domain) calculated based on RRi series from devices with different sampling frequency but with the same sophisticated preprocessing procedures (e.g., detrending methods, resampling, threshold-based artefact correction) may present better agreement than parameters based on counting statistics with only manual artifacts correction procedure. Indeed, we showed better agreement statistics (ICC, LoA) values for time- and frequency-domain parameters in analyzed population of pediatric cardiac patients [36]. Mean absolute percentage difference between linear parameters ranged from 1.5% to 15.8%, and for HRA, from 0.4% to 1100%.

The exploratory nature of the study, the small sample size, heterogenous nature of the study group, the absence of healthy pediatric subjects as a control group, and the study’s focus on only static conditions should be acknowledged as limitations. Limited number of HRA parameters were presented. In future research, it would be interesting to verify agreement of the area asymmetry [37] and the phase asymmetry of the HRV signal [38] between devices with different sampling frequency.

As HRA is a fundamental physiological phenomenon underlying HRV [27], its analysis should be performed before appropriate HRV analysis. The Pneumonitor might be considered valid for recording RRi, which are then processed to calculate time- and frequency domain HRV parameters [36], but also selected HRA parameters, consequently cardiorespiratory coupling, in a group of pediatric cardiac patients in rest condition. It still should be noted that RRi recorded using devices with lower sampling frequency (i.e., with less than 250 Hz) may be not adequate for reliable HRA analysis.

## Figures and Tables

**Table 1:** Results of agreement statistics for HRA parameters calculated based on RRi obtained using ECG and the Pneumonitor.

Parameter	Mean ± SD ECG	Mean ± SD Pneumonitor	Bias (LoA)	ICC (Lower 95% CI)
SD1_d_ [ms]	27 ± 13	29 ± 12	−2 (−5–2)	0.98 (0.86)
SD1_a_ [ms]	25 ± 11	27 ± 10	−2 (−7–2)	0.96 (0.74)
SD2_d_ [ms]	45 ± 15	47 ± 15	−2 (−7–2)	0.98 (0.92)
SD2_a_ [ms]	50 ± 18	52 ± 18	−2 (−7–3)	0.98 (0.90)
C1_d_ or GI	0.54 ± 0.04	0.53 ± 0.04	0.01 (−0.03–0.06)	0.80 (0.50)
C2_d_	0.46 ± 0.05	0.46 ± 0.05	0.00 (−0.03–0.03)	0.96 (0.88)
PI [%]	47 ± 4	46 ± 3	1 (−2–4)	0.88 (0.52)
DR1 [no]	44 ± 17	46 ± 18	−2 (−17–13)	0.91 (0.77)
DR2 [no]	37 ± 11	36 ± 11	1 (−7–9)	0.94 (0.83)
DR3 [no]	8 ± 7	7 ± 6	1 (−3–5)	0.94 (0.78)
DR4 [no]	3 ± 2	2 ± 2	1 (−3–3)	0.75 (0.43)
DR5 [no]	1 ± 1	1 ± 1	0 (−2–2)	0.48 (0.12)
AR1 [no]	39 ± 19	42 ± 18	−3 (−14–7)	0.95 (0.81)
AR2 [no]	37 ± 13	34 ± 11	3 (−6–12)	0.90 (0.67)
AR3 [no]	12 ± 7	10 ± 7	2 (−5–7)	0.90 (0.73)
AR4 [no]	4 ± 4	4 ± 3	0 (−2–3)	0.93 (0.81)
AR5 [no]	2 ± 3	2 ± 3	0 (−2–3)	0.92 (0.79)
NR1 [no]	4 ± 4	12 ± 7	−8 (−17–1) ***	0.34 (0.10)
NR2 [no]	0 ± 0	1 ± 2	−1 (−3–2) *	0.15 (0.23)

GI—Guzik’s index, PI—Porta’s index, *** *p* < 0.001, * *p* < 0.05.

**Table 2:** Correlation coefficient between HRA parameters calculated based on RRi obtained using ECG and the Pneumonitor and RespRate.

Parameter	ECG	PM
C1_d_ or GI	−0.63, *p* = 0.009	−0.32, *p* = 0.230
C2_d_	0.53, *p* = 0.034	0.43, *p* = 0.095
PI [%]	0.78, *p* < 0.001	0.69, *p* = 0.003
DR1 [no]	0.87, *p* < 0.001	0.87, *p* < 0.001
DR2 [no]	0.44, *p* = 0.088	0.41, *p* = 0.111
DR3 [no]	−0.33, *p* = 0.215	−0.44, *p* = 0.091
DR4 [no]	−0.15, *p* = 0.586	−0.23, *p* = 0.392
DR5 [no]	−0.15, *p* = 0.570	−0.04, *p* = 0.897
AR1 [no]	0.89, *p* < 0.001	0.85, *p* < 0.001
AR2 [no]	0.65, *p* = 0.007	0.77, *p* < 0.001
AR3 [no]	−0.25, *p* = 0.346	−0.39, *p* = 0.137
AR4 [no]	−0.55, *p* = 0.027	−0.65, *p* = 0.006
AR5 [no]	−0.62, *p* = 0.010	−0.58, *p* = 0.018
NR1 [no]	0.16, *p* = 0.548	0.26, *p* = 0.336
NR2 [no]	−0.39, *p* = 0.128	0.07, *p* = 0.807

## Data Availability

The raw data supporting the conclusions of this article will be made available by the authors, without undue reservation.
